# Individuals with Le(a+b−) Blood Group Have Increased Susceptibility to Symptomatic *Vibrio cholerae* O1 Infection

**DOI:** 10.1371/journal.pntd.0001413

**Published:** 2011-12-27

**Authors:** Mohammad Arifuzzaman, Tanvir Ahmed, Mohammad Arif Rahman, Fahima Chowdhury, Rasheduzzaman Rashu, Ashraful I. Khan, Regina C. LaRocque, Jason B. Harris, Taufiqur Rahman Bhuiyan, Edward T. Ryan, Stephen B. Calderwood, Firdausi Qadri

**Affiliations:** 1 Centre for Vaccine Sciences, International Centre for Diarrhoeal Disease Research Bangladesh (ICDDR,B), Dhaka, Bangladesh; 2 Division of Infectious Diseases, Massachusetts General Hospital, Boston, Massachusetts, United States of America; 3 Department of Medicine, Harvard Medical School, Boston, Massachusetts, United States of America; 4 Department of Pediatrics, Harvard Medical School, Boston, Massachusetts, United States of America; 5 Department of Immunology and Infectious Diseases, Harvard School of Public Health, Boston, Massachusetts, United States of America; 6 Department of Microbiology and Molecular Genetics, Harvard Medical School, Boston, Massachusetts, United States of America; University of California San Diego School of Medicine, United States of America

## Abstract

**Background:**

Human genetic factors such as blood group antigens may affect the severity of infectious diseases. Presence of specific ABO and Lewis blood group antigens has been shown previously to be associated with the risk of different enteric infections. The aim of this study was to determine the relationship of the Lewis blood group antigens with susceptibility to cholera, as well as severity of disease and immune responses to infection.

**Methodology:**

We determined Lewis and ABO blood groups of a cohort of patients infected by *Vibrio cholerae* O1, their household contacts, and healthy controls, and analyzed the risk of symptomatic infection, severity of disease if infected and immune response following infection.

**Principal Findings:**

We found that more individuals with cholera expressed the Le(a+b−) phenotype than the asymptomatic household contacts (OR 1.91, 95% CI 1.03–3.56) or healthy controls (OR 1.90, 95% CI 1.13–3.21), as has been seen previously for the risk of symptomatic ETEC infection. Le(a–b+) individuals were less susceptible to cholera and if infected, required less intravenous fluid replacement in hospital, suggesting that this blood group may be associated with protection against *V. cholerae* O1. Individuals with Le(a–b−) blood group phenotype who had symptomatic cholera had a longer duration of diarrhea and required higher volumes of intravenous fluid replacement. In addition, individuals with Le(a–b−) phenotype also had lessened plasma IgA responses to *V. cholerae* O1 lipopolysaccharide on day 7 after infection compared to individuals in the other two Lewis blood group phenotypes.

**Conclusion:**

Individuals with Lewis blood type Le(a+b−) are more susceptible and Le(a–b+) are less susceptible to *V. cholerae* O1 associated symptomatic disease. Presence of this histo-blood group antigen may be included in evaluating the risk for cholera in a population, as well as in vaccine efficacy studies, as is currently being done for the ABO blood group antigens.

## Author Summary

Cholera remains a severe diarrheal disease, capable of causing extensive outbreaks and high mortality. Blood group is one of the genetic factors determining predisposition to disease, including infectious diseases. Expression of different Lewis or ABO blood group types has been shown to be associated with risk of different enteric infections. For example, individuals of blood group O have a higher risk of severe illness due to *V. cholerae* compared to those with non-blood group O antigens. In this study, we have determined the relationship of the Lewis blood group antigen phenotypes with the risk of symptomatic cholera as well as the severity of disease and immune responses following infection. We show that individuals expressing the Le(a+b−) phenotype were more susceptible to symptomatic cholera, while Le(a–b+) expressing individuals were less susceptible. Individuals with the Le(a–b−) blood group had a longer duration of diarrhea when infected, required more intravenous fluid replacement, and had lower plasma IgA antibody responses to *V. cholerae* LPS on day 7 following infection. We conclude that there is an association between the Lewis blood group and the risk of cholera, and that this risk may affect the outcome of infection as well as possibly the efficacy of vaccination.

## Introduction

Cholera continues to cause severe diarrheal illness in people with inadequate public health who live in resource-limited settings. Cholera is endemic in countries in Asia and Africa, with new outbreaks reported each year in several countries including, most recently, in Zimbabwe and Haiti [1], [2]. *Vibrio cholerae* O1 is the predominant cause of endemic and epidemic cholera, and this infection is the most common bacterial cause of acute watery diarrhea in adults and children in Bangladesh [3]. There is a close interplay between the organism and the human host in the disease process, and understanding the nature of this interaction is important for understanding pathophysiology, as well as for designing the most appropriate preventive and therapeutic strategies to reduce the morbidity and mortality associated with this infection.

In previous studies, we have analyzed the genes expressed by *V. cholerae* O1 during human infection [4], [5], [6], as well as the human genes expressed in the gut mucosa in response to the infection [7]. These studies have suggested that human innate immune responses are up-regulated in response to *V. cholerae* O1 infection, and these innate immune responses may be important in controlling the disease. Studies of protection from cholera in exposed household contacts indicated that there is a genetic basis for at least some portion of protection from infection [8], [9], and a candidate gene analysis in these contacts identified a polymorphism in the human gene for LPLUNC1, an innate immune response gene, as linked to protection [10], [11].

The other set of human genetic factors that have been studied in relationship to susceptibility to enteric infections are the blood group antigens. For cholera, blood group O has been associated with a lower risk of colonization in exposed household contacts [12] but if colonized, a higher risk of more severe disease [12], [13], [14]. In contrast, blood groups AB or A have been shown to be associated with more severe illness in individuals infected with a related pathogen, enterotoxigenic *Escherichia coli* (ETEC), in children in Bangladesh [15].

Another set of blood group antigens, the Lewis blood group antigens Lewis a (Le^a^) and Lewis b (Le^b^), are carbohydrate antigens related to the ABO blood group antigen that are synthesized in epithelial tissues and adsorbed to the surface of red blood cells [16]; these antigens can also be detected in saliva and other secretions, as well as on cells of mucosal epithelia [17], [18]. The Lewis antigen system has three different phenotypes; Le(a+b−) (these individuals have the nonsecretor phenotype); Le(a–b+), in which a fucosyltransferase converts Le^a^ to Le^b^ (these individuals have the secretor phenotype); or Le(a–b−), in which there is a failure to express either antigen (these individuals can be either secretors or non-secretors) [19]. In a previous study of ETEC diarrhea in Bangladesh, we showed that the approximate proportions of these three phenotypes in the population were: Le(a+b−) 26%; Le(a–b+) 58%; and Le(a–b−) 16% [20]. We also showed that patients with the Le(a+b−) phenotype had an increased risk of having symptomatic ETEC diarrhea compared to the other two phenotypes, particularly if infected with an ETEC strain expressing a CFA/I group colonization factor; this increased risk of symptomatic disease was not seen in patients infected with ETEC expressing other colonization factors, or with rotavirus. Previous studies have suggested that the CFA/I group colonization factors of ETEC bind the Le^a^ antigen on epithelial cells of the small intestine [21]. Conversely, susceptibility to *Helicobacter pylori* infection was higher in Le(a–b+) individuals [22]. In the present study, we analyzed the relationship of Lewis blood group antigen to the risk of symptomatic *V. cholerae* O1 infection in a cohort of patients and their household contacts in Bangladesh, as well as the relationship of Lewis antigen phenotype to severity of and immune responses following disease.

## Materials and Methods

### Study population and sample collection

The study was carried out on patients with cholera presenting to the icddr,b diarrheal disease hospital in Dhaka, Bangladesh. Hospitalized patients with acute watery diarrhea were confirmed by stool culture to be infected with *V. cholerae* O1 as previously described and enrolled on the 2^nd^ day of hospitalization after informed consent [8], [23]. On the same day as patients were enrolled in the study (defined as day 2), field workers enrolled all consenting household contacts of each index patients, defined as individuals who shared the same cooking pot as the index patient for three or more days [23]. Index patients were assessed for other clinical parameter. The type of dehydration status and recovery of patients was assessed by experienced physicans in the icddr,b diarrheal hospital [24]. Household contacts were followed prospectively on study days 2–10, providing daily rectal swabs for cultures for *V. cholerae* O1, as well as giving clinical histories for diarrheal illness. Blood specimens were obtained from index patients and household contacts on study days 2, 7 and 30. Saliva specimens were collected from all participants on study day 2. Saliva specimens were also obtained at one time point from 283 healthy individuals who were from an urban setting and in a similar socio-economic status as the index patients, to determine the distribution of the Lewis blood group antigens in the general population. Blood and saliva samples obtained at day 2 were used for the determination of the ABO and Lewis blood group phenotypes, respectively. Blood samples at each time point were assessed for vibriocidal antibody, and IgG and IgA antibodies against cholera toxin B subunit (CTB) and lipopolysaccharide (LPS) antigens.

### Ethics statement

This study was conducted according to the principles expressed in the declaration of Helsinki. We obtained written consent from each individual prior to participation. Written informed consent was obtained from adults participating in the study. This study was approved by the Ethical and Research Review Committees of the International Centre for Diarrhoeal Disease Research, Dhaka, Bangladesh (icddr,b) and the Institutional Review Board of Massachusetts General Hospital, Boston, MA.

### Confirmation of bacterial strains

For all index cases, stool specimens were cultured on taurocholate-tellurite gelatin agar (TTGA) plates for isolation of *V. cholerae*. After overnight incubation of plates, specific monoclonal antibodies were used to detect *V. cholerae* O1, and the Ogawa and Inaba serotypes by slide agglutination test [25], [26]. Rectal swabs from household contacts were collected in Cary-Blair transport media, taken to the icddr,b, and cultured on TTGA followed by colony identification as above. Some specimens were also enriched in alkaline peptone water for 4 hours prior to culturing [3].

### Blood group ABO typing

For ABO blood group typing, a slide agglutination test was carried out according to the manufacturer's instruction (Biotec laboratories, UK).

### Lewis blood group typing

Lewis blood group phenotype was determined using saliva samples and a dot blot immunoassay procedure [20], [27]. For this purpose, 2 µl of saliva were applied to nitrocellulose membrane strips and allowed to dry. After blocking with 1% bovine serum albumin, mouse monoclonal anti-Le^a^ and anti-Le^b^ antibodies (Abcam, Cambridge, UK) were added and the strips were incubated for 30 min at room temperature with gentle shaking. The strips were then washed and incubated with secondary, horseradish peroxidase-conjugated antibody for another 30 min. After washing, the strips were developed with 4-chloro-1-naphthol and 3% hydrogen peroxide. A specimen was considered positive when a dark black spot appeared on the membrane.

### Immunological assays

Vibriocidal antibody assays were performed using guinea pig complement and the homologous serotype of *V. cholerae* O1 isolated from the patient, either El Tor Ogawa (strain 25049) or El Tor Inaba (strain T-19479) as previously described [28]. The vibriocidal titer was defined as the reciprocal of the highest plasma dilution resulting in >50% reduction of the optical density compared to that of control wells without plasma. Seroconversion was defined as a 4-fold or higher increase in vibriocidal titer after infection. Plasma IgG and IgA antibodies specific to CTB and LPS were measured by kinetic ELISA procedure as previously described [29], [30]. In brief, 96-well microtiter plates were coated with either purified *V. cholerae* O1 LPS (250 ng/well), or GM1 ganglioside (100 ng/well) followed by recombinant CTB (50 ng/well). Plates were incubated with diluted patient sera (1∶50 for LPS ELISA and 1∶200 for CTB ELISA), washed, and horseradish peroxidase-conjugated secondary antibodies to human IgG or IgA (Jackson Laboratories, Bar Harbor, Maine) were applied in separate wells. Plates were developed using 0.1% orthophenylene diamine (Sigma, St. Louis, Missouri) in 0.1 M sodium citrate buffer with 0.1% hydrogen peroxide, and optical densities (OD) were read kinetically at 450 nm for 5 minutes at 19-s intervals and results expressed as milliabsorbance/min (mAb/min). ELISA values were calculated by taking the ratio of the value obtained for the test specimen to that obtained for the positive control specimen and multiplying by a factor of 100. Pooled plasma was prepared using specimens from convalescent stage cholera patients from an earlier study [29].

### Statistical analyses

Statistical analyses were performed on SPSS 17.0 and SigmaStat 3.1 programs. Graphs were prepared using the Prism 5.0 software (GraphPad Software Inc.). The association between Lewis blood groups and symptomatic cholera was assessed by the chi-square test. Associations were also carried out by calculating the odds ratio (OR) with 95% confidence intervals (CI) using EpiInfo 3.3.2. The Wilcoxon signed rank test was used to compare immune responses of patients on different follow-up days and the Mann-Whitney U test was used for comparison among different groups. All reported P values are two tailed and significance was defined as P<0.05.

## Results

### Demographic features and clinical outcomes of patients and household contacts

Ninety five cholera patients, 144 household contacts, and 283 healthy controls were enrolled in the study overall (Table 1). The median age of the patients enrolled in the study was 28 years while that for the household contacts was 23 years and of healthy controls was 18 years. The controls were younger than the patients and healthy contacts (P<0.001). The proportion of males and females in each group was not significantly different. Thirty five household contacts had positive rectal swabs for *V. cholerae* O1 during follow up and of these, 20 had diarrhea and were considered to have symptomatic cholera; these 20 were excluded from the analysis of Lewis blood group types in contacts. Among the index patients, 80% (76/95) were infected with the Ogawa serotype of *Vibrio cholerae* O1 and 20% (19/95) were infected with *Vibrio cholerae* O1 Inaba. At the time of hospitalization, 92% (87/95) of the index patients were severely dehydrated. The average duration of diarrhea for all index patients was 57 hours and patients received on average 7.5 liters of intravenous rehydration.

**Table 1:** Demographic characteristics of study participants.

Variable	Patients (n = 95)	Household contacts (n = 144)	Healthy Controls (n = 283)
Median age (range)	28 (4–59 yr)	23 (3–60 yr)	18 (4–49 yr)
% Female	42	50	53
No. rectal swab positive	95	35	-
No. with severe dehydration	87	-	-

### Distribution of ABO blood group in the study participants

Among the 95 index patients, 43% were blood group O positive, 34% were blood group B, 19% were blood group A, and 4% were blood group AB. The asymptomatic contacts had a similar distribution of ABO blood groups (47%, 27%, 18% and 8% respectively). We did not determine the ABO blood group of individuals enrolled as healthy controls but this has been done in earlier studies [12], [46]. The distribution of ABO blood group in a similar setting in Bangladesh has been shown to be for the O∶A∶B∶AB groups to be 28%∶23%∶38%∶11% respectively [46].

### Distribution of Lewis blood group phenotypes and symptomatic cholera

**Figure 1:**
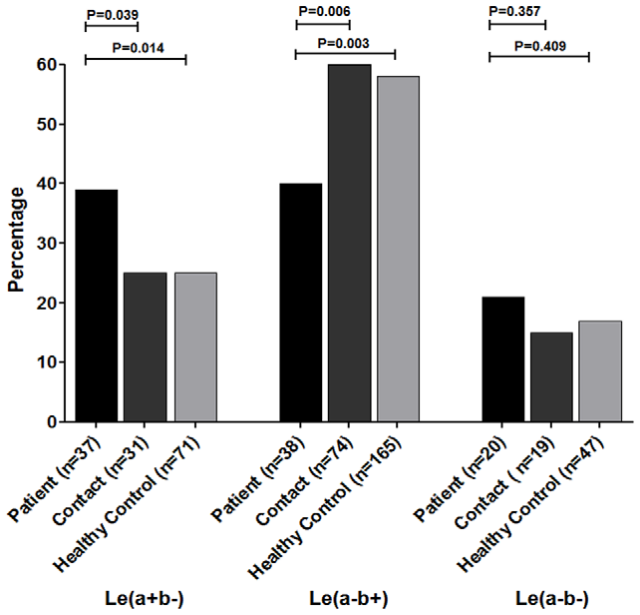
Association between Lewis blood group and symptomatic cholera infection. Lewis blood group phenotype was determined using dot blot immunoassay procedure from saliva samples. The chi-square test was used to compare the distribution of Lewis blood types in different populations, i.e. patients, contacts and healthy controls.

In the 522 study participants overall, 28% were Le(a+b−), 55% were Le(a–b+), and 17% were of the Le(a–b−) blood group phenotype, very similar to the proportions shown in this population previously [15]. In comparing the Lewis blood group phenotype distributions between patients symptomatic with cholera compared to asymptomatic household contacts and healthy controls, patients were enriched for the Le(a+b−) phenotype (39%) and had fewer individuals in the Le(a–b+) phenotype (40%); both these were significantly different than the frequencies of these phenotypes in asymptomatic contacts and healthy controls (Figure 1). In contrast, the distribution of the three phenotypes in asymptomatic contacts and healthy controls were virtually identical to each other and to the overall population. The Le(a+b−) blood group phenotype was significantly associated with having symptomatic cholera, as compared to the household contacts who were asymptomatic (OR 1.91, P = 0.039, 95% CI 1.03–3.56) or to the healthy controls (OR 1.90, P = 0.014, 95% CI 1.13–3.21). Similarly the frequency of Le(a–b+) was lower in patients than the contacts (OR 0.45, P = 0.006, 95% CI 0.25–0.81) or healthy controls (OR 0.48, P = 0.003, 95% CI 0.29–0.79). No relationship was found between the presence of Le(a–b−) blood group phenotype and susceptibility to cholera comparing the three groups of study participants (Figure 1).

### Association of cholera with Lewis blood group phenotypes within individual ABO blood groups

**Figure 2:**
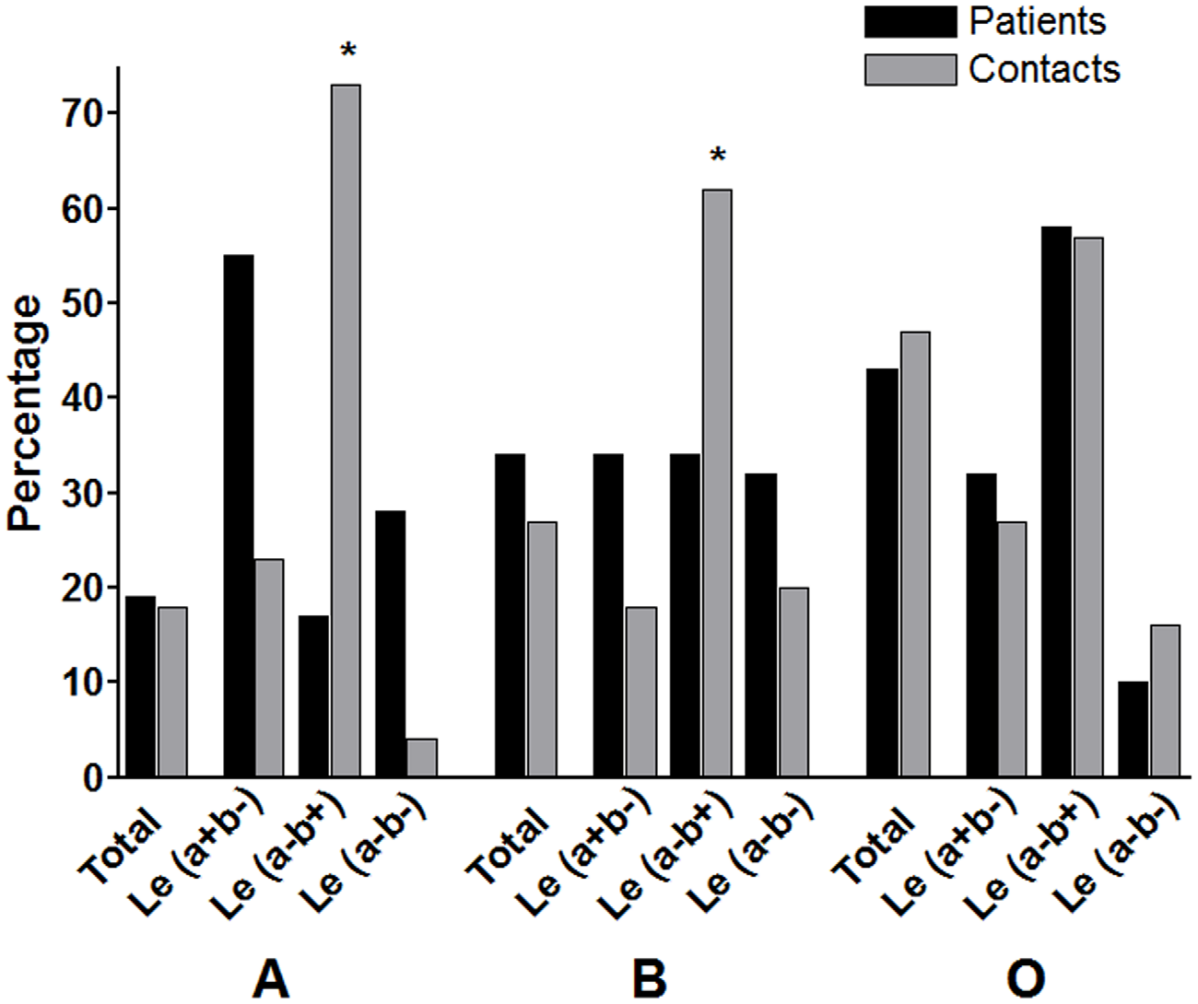
Association between ABO and Lewis blood group and cholera infection. Statistical analyses were done to determine the relationship of cholera with Lewis blood group types in each ABO group phenotype individually.

We also analyzed the presence of different combinations of Lewis blood group antigens and ABO blood groups in relation to susceptibility to cholera (Figure 2). In individuals with the A blood group, the Le(a–b+) phenotype was less common in patients than household contacts (P = 0.001), while the percentage of the Le(a+b−) phenotype in patients trended toward being higher than in contacts (P = 0.071), as seen for the group overall. Similarly, in individuals with blood group B, we found a lower frequency of the Le(a–b+) phenotype in patients compared to contacts (P = 0.048), as for the analysis in the study population overall. However, we did not find any significant associations of Lewis blood group antigens and symptomatic cholera in patients with blood group O. The small number of individuals with blood group AB (n = 14) prevented any firm conclusions for this blood group.

### Association of Lewis phenotypes with immunologic responses to cholera in patients

**Figure 3:**
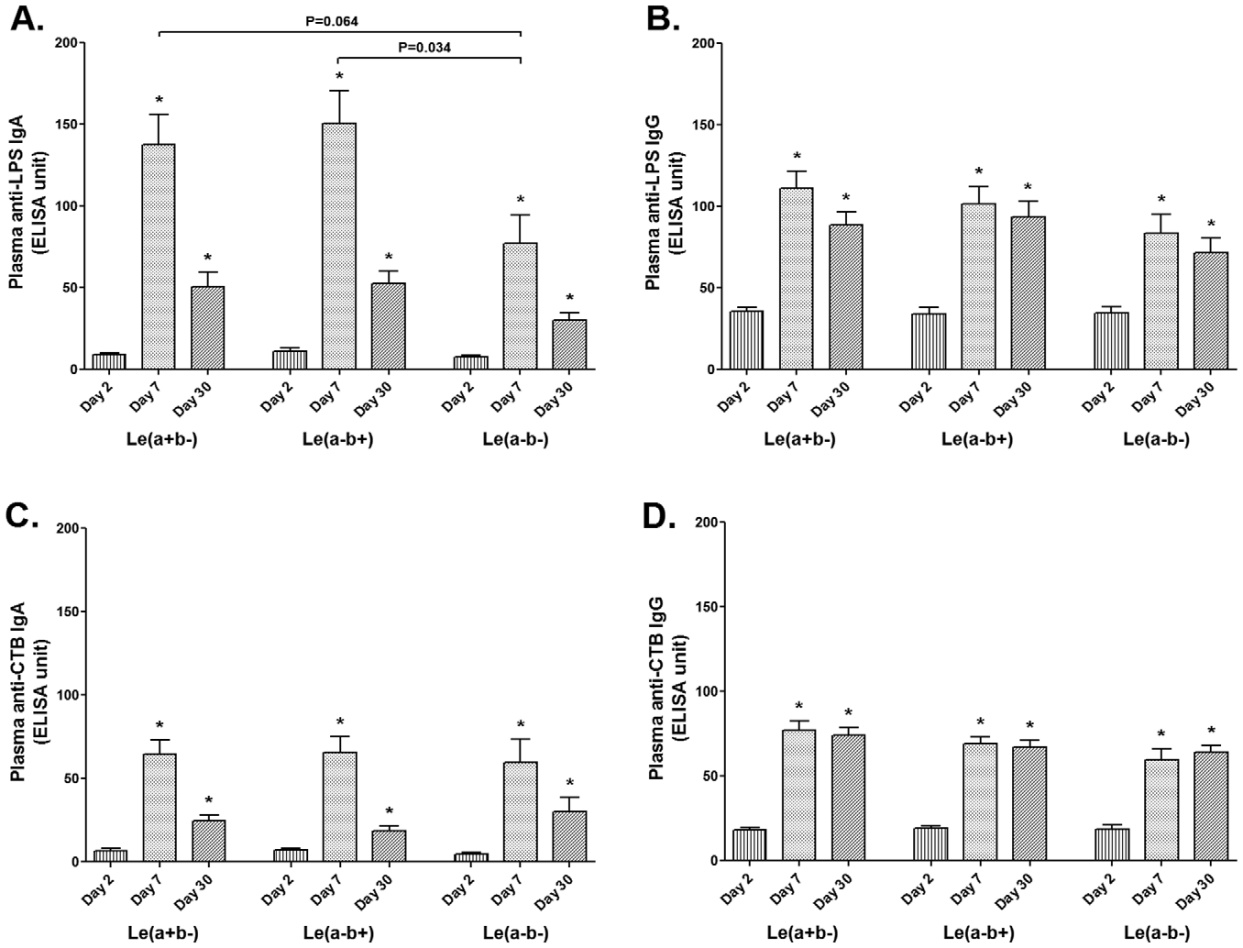
Association between Lewis blood group and immune responses following cholera. The Signed rank test was used to compare antibody responses on different days. The Mann Whitney U test was used to compare immunologic responses in patients of different Lewis blood groups. Asterisks indicate significantly higher responses at convalescence (day 7 and day 30) compared to that in acute stage (day 2), using a paired t-test. Brackets denote statistically significant lower LPS-specific IgA responses on day 7 in patients with the Le(a–b—) phenotype compared to the other two groups.

We next assessed whether there were any differences in immune responses to *V. cholerae* O1 infection in individuals with the various Lewis blood group phenotypes. We found no differences in plasma vibriocidal titers on days 2, 7, or 30 between index patients with the different Lewis blood group phenotypes (data not shown). In analyzing IgG and IgA responses to CTB and LPS, we also found no differences in either IgG or IgA responses to CTB or the IgG responses to LPS (Figure 3). However, patients with the Le(a–b−) phenotype developed significantly lower LPS IgA responses on day 7 compared to the individuals with the Le(a–b+) phenotype (P = 0.034); there was a trend of lower responses when compared to those of the Le(a+b−) phenotype (P = 0.064). The responses in patients in the different Lewis groups were comparable by day 30 (Figure 3A).

### Association of Lewis blood group antigen with severity of cholera in index patients

Because of the differences in IgA responses to LPS between individuals with the different Lewis blood group antigens, we also compared the severity of cholera in these three groups. There were no differences between the groups in the time between onset of symptoms and presentation to the icddr,b, in the duration of diarrhea pre-hospitalization, in the use of antibiotics prior to presentation, or in the average ORS consumed before presentation (data not shown). However, once hospitalized, patients with the Le(a–b+) phenotype required significantly less intravenous fluids compared to individuals with either the Le(a+b−) (P = 0.017) or Le (a–b−) (P<0.001) phenotypes (Table 2). In addition, patients with the Le(a–b−) phenotype had a significantly longer duration of diarrhea than did the patients of Le(a–b+) or Le(a+b−) groups (P = 0.012 and 0.017, respectively), correlating with their increased need for intravenous hydration (Table 2).

**Table 2:** Association of Lewis blood group with clinical characteristics of hospitalized cholera patients.

Variable	Lewis Phenotype	
**Number with severe dehydration**	Le(a+b−)	32/37
	Le(a–b+)	37/38
	Le(a–b−)	18/20
**Average duration of diarrhea**	Le(a+b−)	54 hr
	Le(a–b+)	53 hr
	Le(a–b−)	73 hr*
**Average quantity of IV fluid required**	Le(a+b−)	8.3 L
	Le(a–b+)	5.6 L*
	Le(a–b−)	10.7 L

*Significantly different (P<0.05) from other groups by Rank sum test.

## Discussion

In this study, we investigated the relationship of the Lewis blood group antigens with susceptibility to cholera and to the clinical course of the illness. We determined the Lewis blood group using saliva samples, which have been previously shown to be concordant with typing carried out using blood specimens [20]. The overall ABO and Lewis blood group antigen distribution was similar to that seen in other studies carried out recently in Bangladesh [15], [20].

Our first finding in this study was that individuals with the Le(a+b−) phenotype were more likely to get symptomatic cholera compared to the other two groups, suggesting that this Lewis blood group may be associated with an increased risk of being colonized with *V. cholerae* O1 or if colonized, of becoming symptomatic. This same Lewis blood group has previously been shown to increase susceptibility to symptomatic ETEC infection if the organism is expressing a CFA/I group colonization factor [15], [20]. Interestingly, in looking at the inter-relationship between risk of symptomatic cholera and both ABO and Lewis blood groups, the increased risk of symptomatic infection in Le(a+b−) individuals was only seen in individuals who had the A or B blood groups, and not blood group O. Since blood group O is itself a risk factor for more severe cholera, it is possible that an effect of the Lewis blood group types was not evident because of the higher risk of symptoms already conferred by the O blood group. Our second finding, that individuals with the Le(a–b+) phenotype required less intravenous fluids following hospitalization than individuals of the Le(a+b−) phenotype, is also consistent with a difference in severity of cholera, once it occurs, between these two Lewis blood groups.

Our third finding was that individuals in the Le(a–b−) phenotype admitted to the icddr,b with cholera required the most intravenous fluids and had the longest duration of diarrhea, suggesting that this phenotype, while not over-represented in patients with cholera, was associated with an increased severity of disease once it occurs. We observed that there was a trend of susceptibility to cholera for those in the Le(a–b−) group also, but possibly because of the small sample size, the analysis did not reach statistical significance. The fourth finding in our study was that individuals with the Le(a–b−) phenotype had reduced IgA responses to LPS compared to individuals in the other two phenotypes although comparison with Le(a+b−) did not reach significance. The plasma level of IgA reactive to LPS on exposure is correlated with protection from subsequent infection with *V. cholerae* O1 [8]. Index patients in the different Lewis blood group types did not have any significant differences in baseline IgA reactive to LPS, just a difference in magnitude of response at day 7. It is not known if this reduced magnitude of LPS-specific IgA on day 7 is associated with the longer duration of diarrhea and therefore higher requirement for intravenous fluid in this subgroup of individuals; the differences in LPS-specific IgA between groups was not evident by day 30 post infection.

Histo-blood group antigens can predispose individuals to genetic, metabolic, and infectious diseases, including enteric illnesses. Blood group antigens are fucosyloligosaccharides that are expressed in the gut epithelium and hence can act as potential receptors for enteric pathogens [22], [31]. This can make individuals of one blood group type more susceptible to a particular pathogen compared to individuals expressing other blood group antigens. Another mechanism of association with disease is that soluble forms of these antigens can be secreted into the gut lumen and might prevent colonization of pathogens by competitive neutralization [32]. Earlier studies have shown that individuals with Lewis blood group Le(a–b+) are at higher risk for colonization by *H. pylori* [22], [33]. *Campylobacter jejuni* binds to intestinal H (blood group O) antigen and it has been shown that fucosyloligosaccharides in human milk can inhibit binding and infection by this organism [34]. Norovirus has been particularly well studied for the association with blood group antigens. This pathogen binds specifically to A, H and difucosylated Lewis antigens but not to the B antigen [35], which is supportive of earlier studies in which it was shown that individuals with O blood group were more prone to Norovirus infection, while it was less likely in individuals of the B blood group [36].

Susceptibility to *V. cholerae* infection is believed to result from a combination of factors including exposure, lack of immunity on encountering the organism [8], [12], nutritional deficiencies [37], [38], and human genetic polymorphisms [11]. Individuals with blood group O are at a higher risk of developing severe cholera than those with other blood groups [12], [13], [14], [39]. It is hypothesized that this may have resulted in a selective pressure for human genetic evolution that may explain the lower prevalence of the O blood group in cholera endemic regions such as Bangladesh and other areas near the Ganges delta [40]. In the present study, we did not find any association of cholera with the presence of specific ABO blood groups, perhaps related to our smaller sample size. However, we did find a strong association with the Lewis blood group phenotypes, although the reasons behind this observation are not yet defined. Two possibilities are that, as for ETEC, the Le^a^ antigen may act as a receptor on mucosal epithelia for a cholera ligand. However, Le(a+b−) individuals are also non-secretors, so it is possible that the association of this phenotype with symptomatic cholera is intertwined with the non-secretor status rather than the Le^a^ and Le^b^ antigens themselves.

In contrast to cholera, individuals with blood groups A and AB are at higher risk for ETEC infection [15], [41], [42]. On the other hand, for the Lewis blood group antigens, individuals with the Le(a+b−) phenotype are more susceptible to both symptomatic cholera as well as ETEC infection. The distribution of Lewis blood group phenotypes in this study was different from that reported in a Caucasian population in a cholera non-endemic area, but similar to populations studied in India and Africa, where cholera is endemic [19], [43], [44]. However, unlike the relationship between ABO blood group and cholera, the Lewis blood group phenotypes have apparently not been selected for by cholera, as the more susceptible type, Le(a+b−), is more frequent in the endemic areas than in those areas without endemic cholera [19], [43], [44]. Perhaps selective pressure for the Lewis blood group antigens is weaker than for the ABO blood group system, and the endemicity of cholera and ETEC diarrhea in settings such as Bangladesh may be partially explained by the increased presence of the Le(a+b−) phenotype.

Factors influencing susceptibility to cholera may also play a role in responses to cholera vaccines. For example, in a large scale field trial conducted in Matlab, Bangladesh of the role of ABO blood group and efficacy of an oral, killed cholera vaccine, there was substantially lower protection in recipients who were blood group O [45]. We have also shown previously that in Bangladeshi children receiving a live, oral attenuated cholera vaccine, Peru 15, the frequency of serological responders was higher in children of the A blood group compared to the O blood group [46]. Thus, the ABO blood group system is a potential factor that may affect vaccination efficacy in different settings, and this factor has now been included in the assessment of ongoing cholera vaccine trials. In the present study, we found that individuals who were negative for both Lewis antigens ‘a’ and ‘b’ had impaired LPS-specific IgA responses on day 7 compared to individuals with Le (a–b+) Lewis antigen phenotypes. This suggests that inclusion of the Lewis blood group should be considered in cholera vaccine efficacy trials in the future as well as the ABO blood group types.
